# Dysnœa on Falling Asleep

**Published:** 1887-02

**Authors:** E. W. Berridge

**Affiliations:** London


### DYSPNŒA ON FALLING ASLEEP.

E. W. Berridge, M. D., London.

In reply to some questions concerning his paper on *Cad.-sulph.*, Dr. Kent has sent me such valuable information that I think it should not be lost to the profession. He writes: "Loss of breath on going to sleep should be *Carb.-v.*, but *Carbo an.* has in my hands cured it. I have seen several cases of *Opium* poisoning when the symptom was present. I have cured it with *Opium.* I have verified it in both *Grindelia robusta* and *G. squarrosa.* You can also add *Cad.-s.* to your list. *Cad.-s.* has interrupted breathing during sleep, and from this I have dilated the symptom clinically to 'stops breathing on going to sleep, wakes up suffocating.' *Opium* stops breathing on going to sleep, and must be shaken to start him to breathing. I saw a patient over-sensitive to *Opium* once who had this symptom from one-tenth of a grain." In a former number of The Homœopathic Physician I gave a list of medicines which had produced or cured this important symptom. The full list is now *Amm.-carb., Ant.-tart., Arum.-triph., Badiaga, Bryon., Cadm.-s., Carb.-an., Carb.-veg., Grindelia robusta, Graphit., Lachesis, Nux-mosch., Opium, Ranunc.-bulb., Grindel. squarrosa.* The misprint of *Carb.-an.* for *Carb.-veg.* occurs in Jahr's German *Repertory,* though, as it happens, it now belongs to both.

